# Health literacy and medication adherence in adults from ethnic minority backgrounds with Type 2 Diabetes Mellitus: a systematic review

**DOI:** 10.1186/s12889-024-20734-z

**Published:** 2025-01-20

**Authors:** Jinal Parmar, Aymen El Masri, Freya MacMillan, Kirsten McCaffery, Amit Arora

**Affiliations:** 1 School of Health Sciences, Western Sydney University, Campbelltown, NSW 2560 Australia; 2 Health Equity Laboratory, Campbelltown, NSW 2560 Australia; 3 Translational Health Research Institute, Western Sydney University, Campbelltown, NSW 2560 Australia; 4 Office of the Deputy Vice-Chancellor (Research, Enterprise and International), Western Sydney University, Penrith, NSW 2751 Australia; 5 Diabetes Obesity and Metabolism Translational Research Unit, Western Sydney University, Campbelltown, NSW 2560 Australia; 6 Sydney Health Literacy Lab, Sydney School of Public Health, The University of Sydney, Camperdown, NSW 2050 Australia; 7 Discipline of Child and Adolescent Health, The Children’s Hospital at Westmead Clinical School, Faculty of Medicine and Health, The University of Sydney, Westmead, NSW 2145 Australia; 8 Oral Health Services, Sydney Local Health District and Sydney Dental Hospital, NSW Health, Surry Hills, NSW 2010 Australia

**Keywords:** Health literacy, Medication adherence, Ethnic minority, Type 2 diabetes mellitus, Systematic review

## Abstract

**Background:**

For people living with Type 2 Diabetes Mellitus (T2DM), achieving optimal health outcomes requires optimal self-management and adherence to medical treatment. While some studies suggest an association between poor medication adherence and lower levels of health literacy, the evidence for this association remains inconclusive. This systematic review aimed to synthesise the evidence on the association between health literacy and medication adherence among adults from ethnic minority backgrounds living with T2DM.

**Methods:**

Medline (Ovid), The Cochrane Library, Embase (Ovid), PsycInfo (EBSCO), and the Cumulative Index to Nursing and Allied Health Literature (CINAHL) (EBSCO) were searched systematically for peer-reviewed literature, published until January 2024. Studies were included in this review if they assessed health literacy and medication adherence among ethnic minority people with T2DM. Two reviewers independently screened and selected the studies, extracted data from the included articles, and assessed the methodological quality of the studies. The methodological quality and bias in designing, conducting, and analysis of each study were evaluated using a standardised JBI critical appraisal tool.

**Results:**

Of the total 6,318 identified studies, seven studies were included in the review. The total participant sample sizes across these studies varied from 53 to 408 participants. All included studies incorporated cross-sectional design for the research, with the majority conducted in the USA. Of the seven unique studies, only one study observed a significant association between health literacy and medication adherence among people from an ethnic minority background.

**Conclusions:**

Evidence on the association between health literacy and medication adherence in ethnic minority adults with T2DM is weak and inconsistent. To understand this association more clearly in ethnic minority populations and to address the disparities in cultural and linguistic considerations, well-designed studies are required.

**Trial registration:**

This review is registered with PROSPERO (CRD42022328346).

**Supplementary Information:**

The online version contains supplementary material available at 10.1186/s12889-024-20734-z.

## Background

The term health literacy was first introduced in 1970 and the concept has evolved and been redefined continuously since [1]. A recent systematic review [1] exploring the meaning of health literacy has defined it as the ‘ability of an individual to obtain and translate knowledge and information in order to maintain and improve health in a way that is appropriate to the individual and system contexts’(P.7). This ability helps people to make appropriate healthcare decisions, understand health risk behaviours, enhance health outcomes, and reduce the cost of care in ways that benefit their health [2, 3]. Health literacy is now recognised as a social determinant of health, which is responsive to change using interventions [4].

Low levels of health literacy are directly related to poor health outcomes [5], higher use of emergency services, higher rates of hospitalisations, lower rates of utilising preventive services, increased likelihood of making medication errors [2, 6], poorer understanding of medication instructions [2, 5], increased cost of health care [3], poorer ability to self-care, and a higher risk of mortality [7]. Health literacy skills are influenced by various demographic and social factors including education, socioeconomic status, occupation, income, social support, age, cultural background/ethnicity, language, gender, disability, and race, which act as antecedents of health literacy [2, 8, 9].

Individuals with low education, low income, low socioeconomic background and belonging to ethnic minority backgrounds are at a higher risk of having low health literacy levels [10] and often experience barriers in accessing health care [8]. Lack of cultural competency among healthcare professionals is also a barrier for individuals from ethnic minority backgrounds to access and utilise healthcare services [8, 11]. It is essential to recognise that these barriers exacerbate health inequities. The link between minority status and health literacy indicates that the most disadvantaged groups often have weaker health-related skills which lead to health disparities [10]. Addressing these disparities is crucial for achieving greater health equity and improving the health status of disadvantaged populations.

Ethnic minority groups are at a higher risk of chronic conditions as migration-related stress and changes in lifestyle are critical risk factors in developing chronic conditions, such as Type 2 Diabetes Mellitus (T2DM) and hypertension, in comparison to non-ethnic minority populations [11]. T2DM is a chronic condition defined as having high levels of glucose in the blood, also known as hyperglycaemia [12]. It is a major contributor to other health-related complications such as renal disease, cardiovascular disease, stroke, visual impairment, and lower limb amputation [11, 13]. T2DM is the ninth leading cause of mortality worldwide, attributing to around 1 million deaths annually [14]. In 2021, around 529 million people were affected by T2DM, and the cases are projected to increase to 7,079 per 100,000 globally by 2030 [15]. Major risk factors for T2DM include obesity, physical inactivity, poor diet, ageing, cardiovascular disease, high blood pressure, impaired glucose tolerance, and gestational diabetes [11, 16]. Among ethnic minority populations, leading risk factors that increase the risk of developing T2DM include immigration, genetics, socioeconomic status, and socio-cultural factors [11, 17].

T2DM can be managed by making lifestyle changes including healthy eating habits and daily physical activity [16]. Alongside lifestyle modification, oral anti-diabetic medications, and insulin play a crucial role in diabetes management, consequently, adherence to medications is important in achieving desired health outcomes [9, 18–20]. Medication adherence is defined as a process in which patients take their medications as prescribed by their healthcare providers [21]. Suboptimal adherence may lead to treatment failure, adverse health outcomes, and undesired medical expenses [22, 23]. Specific to T2DM, improvement in adherence to oral anti-diabetic medications results in better glycaemic control, decreased long-term complication development, and a reduction in health care costs [23, 24]. It is evident that T2DM puts a considerable burden of disease management on patients [5]. Other than cognitive factors such as health literacy, there are demographic factors such as age, gender, race, education level, and income that also have an impact on diabetes medication adherence [25, 26]. Moreover, in ethnic minority groups, low health literacy levels lead to an incomplete understanding of disease and treatment regimens [27], high chances of misinterpreting medication labels [5] which may influence their attitudes towards medications for diabetes management [26], which may result in medication non-adherence [27]. Identified factors that affect medication adherence include lack of knowledge of clinical indication; treatment duration or administration timing; lack of knowledge of the consequences of adherence or non‐adherence; and extent of knowledge on medication side‐effects [28].

The broad range of studies conducted across different countries, ages, and with patients with different health conditions have reported that health literacy has a direct impact on medication adherence and have found a statistically significant and positive association between health literacy and medication adherence [29–40], while other studies have reported a positive association between health literacy and medication adherence, but do not support a strong association [41–44]. Yet, there are studies and systematic reviews that have found that there is no direct association between health literacy and medication adherence [25, 41, 45, 46], but found a significant moderator impact of low health literacy on medication adherence by influencing patients’ medication beliefs [30, 41, 46, 47]. Most studies have generated conflicting and inconsistent results which may be due to such associations only observed for some health conditions and not others [5, 41]. A review of systematic reviews [48] examining the association between health literacy and adherence suggested that evidence on the relationship between health literacy and adherence is relatively weak.

Several scoping searches of the literature were conducted to identify existing systematic reviews that have focused on the association between health literacy and medication adherence in patients with diabetes. Thirteen systematic reviews were found from the search [5, 9, 25, 46, 49–53], but a thorough assessment revealed a gap in knowledge. None of the systematic reviews focused on people from ethnic minority backgrounds with T2DM (see Appendix 1). Moreover, three reviews incorporated the evidence from observational and interventional studies without distinguishing the results based on study design. Most of these reviews conducted searches covering articles published up until 2016, except for one that covered articles published up until 2020 [5]. Furthermore, the methodological quality of previous systematic reviews was appraised using the AMSTAR 2 tool by two independent reviewers (AA and JP), and most reviews were rated as “critically low”. These gaps highlight the need for a high-quality systematic review to examine the association between health literacy and medication adherence focusing on ethnic minority population with T2DM. Therefore, this systematic review aimed to examine the evidence on the association between health literacy and medication adherence in people from ethnic minority backgrounds living with T2DM. This body of work may be used to inform future interventions for improving medication adherence in adults from ethnic minority backgrounds with T2DM.

## Methods

**Fig. 1:**
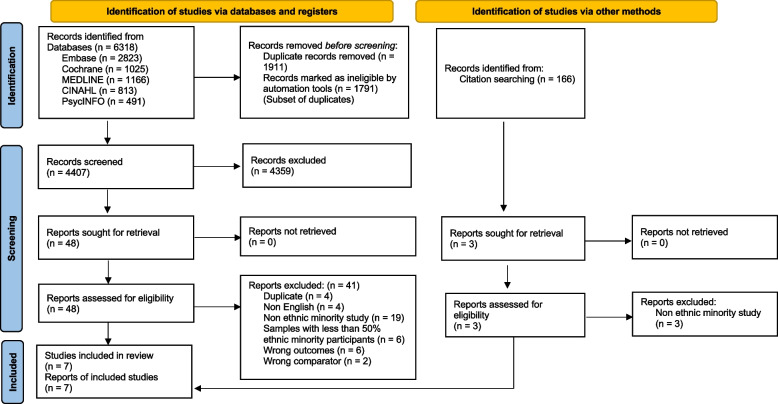
PRISMA 2020 flow diagram for new systematic reviews which include searches of databases and registers [54]

This review is reported using the Preferred Reporting Items for Systematic Reviews and Meta-Analysis (PRISMA) 2020 guidelines (Fig. 1) [54]. The protocol of this systematic review was registered with PROSPERO International Prospective Register of Systematic Reviews (2022 PROSPERO CRD42022328346) [55]. The protocol paper of this systematic review was published on the online digital repository platform—Figshare [56].

### Inclusion criteria

The Population, Exposure, and Outcome (PEO) criteria [57] (Appendix 2) was used to define our inclusion/exclusion criteria. Studies were included if they:

- Measured health literacy and medication adherence using either a subjective measurement tool or objective measurement tool, or both
- examined the association between health literacy and medication adherence
- included samples of at least 50% or more from ethnic minority populations—term used for included population in the study can be ethnicity; ethnic minority; or minority ethnic groups; or race; or specific names of cultural backgrounds such as African, Asian, and Hispanic
- focused on T2DM and incorporated any study design
- were published in the English language
- were available as full-text journal articles
- were published from inception (earliest available date) until January 2024

### Exclusion criteria

Studies were excluded if they:

- focused on Type 1 Diabetes Mellitus or Gestational Diabetes
- were review articles, editorials, commentaries, or conference abstracts
- lacked sufficient data on health literacy or medication adherence measures
- focused on concepts related to health literacy and medication adherence but do not directly measure and analyse the association

### Information sources

The following databases were searched: MEDLINE (Ovid), The Cochrane Library, The Cumulative Index to Nursing and Allied Health Literature (CINAHL) (EBSCO), PsycInfo (EBSCO), and Embase (Ovid). The initial systematic search was performed on 22nd April 2022, an updated search was performed on 23rd Jan 2024. Further, reference lists of all included articles were screened, and a manual search was performed for previous systematic reviews.

### Search strategy

The Population, Exposure of Interest and Outcome (PEO) criteria (Appendix 2) was used to devise the review question and relevant search terms. Search terms included three key terms: health literacy, medication adherence, and T2DM. Ethnic minority search terms were not included because of the broad number of terms used to define ethnic minority people globally. Therefore, people from ethnic minority backgrounds within studies was included as an inclusion criterion during the assessment of articles for full-text eligibility. A combination of keywords and Boolean operators, truncations, phrase searching, and subject headings were used in the search strategy in consultation with a Health Sciences librarian. The search strategy was pre-tested in the MEDLINE (Ovid) database and subsequently tailored to suit the various functions and operators associated with each database. The search strategy from MEDLINE (Ovid) is provided in Appendix 3. Further, the authenticity of the search strategy was tested by searching the inclusion of previously conducted relevant systematic reviews within the final search results.

### Study selection process

Studies identified through the five electronic databases and manual searches were uploaded to the reference manager software Covidence, and duplicates were removed. Articles that met the inclusion criteria were retrieved as full-text and were imported into Covidence for review. Two reviewers (JP and AA) independently assessed the full-text articles for eligibility. Any disagreements were resolved through discussion including a third reviewer (FM or AE). The process of study selection was carried out in accordance with the PRISMA 2020 checklist and presented as a flow diagram (Fig. 1).

### Data collection process and data items

A standardised data extraction form was developed and pilot-tested independently by two reviewers (JP and AA). The data that was extracted included first author, publication year, title, country of study, study design, study setting, sample size, participant characteristics, inclusion criteria, data collection methodology, statistical method used for analysis, study outcomes (association between health literacy and medication adherence), confounders identified and adjusted for, and limitations. Data extraction was conducted primarily by two reviewers (JP and AA) independently. FM and AE provided feedback and resolved disagreements if any. For missing data and/or uncertainties, the study authors were contacted for further information a maximum of three times.

### Assessment of methodological quality

Two independent reviewers (JP and AA) assessed the methodological quality of all the eligible studies before their inclusion in the systematic review. A standardised Joanna Briggs Institute (JBI) critical appraisal tool [58] was utilised to evaluate the methodological quality in relation to bias in designing, conducting, and analysis of the study. The JBI critical appraisal tool evaluates studies based on criteria, such as having a clear inclusion criteria, detailed descriptions, validated measurements, confounding factor management, and appropriate statistical analyses, ensuring high methodological quality [58]. No studies were excluded based on risk of bias assessments. Any disagreement between the two reviewers was resolved through discussion or by including reviewer FM and AE where required.

### Data synthesis

The included articles were reviewed in detail and categorised into current evidence on the association between health literacy and medication adherence in adults from ethnic minority backgrounds with T2DM. The included articles were assessed independently by two appraisers (JP and AA) and the results were reported descriptively for the present systematic review.

## Results

### Study selection

The initial search yielded 6,318 records, which was reduced to 4,407 unique records after removing duplicates and reference searching yielded 166 records. Of the 4,573 studies (total of 4,407 and 166) screened against title and abstract, 51 studies were selected for full-text review. Upon full-text assessment of these studies, seven unique studies were deemed eligible for inclusion in this review (Fig. 1) and 44 studies (41 of databases search and 3 of citation search) were excluded because they did not meet the inclusion criteria (Appendix 7).

### Study characteristics

All seven included studies employed a cross-sectional design. The total participant sample sizes across these studies varied from 53 to 408 participants. The included studies were published between 2006 to 2022. The majority of these studies included adults who were aged 18 years or older, but there was one study that only enrolled people aged 30 years or older [59]. The majority of studies were conducted in the United States, except for one study which was conducted in Canada [60]. Most studies included participants who had a sufficient understanding of the English language and were able to communicate in English. However, one study included only Spanish-speaking Hispanic patients who were not fluent in speaking English [61] and two studies provided participants with the option to choose between English and Spanish as their language for data collection [59, 62]. Most of the studies were conducted in primary care clinics and community health centres with the exception of one virtual study, which utilised social media for participant recruitment and data collection [60] (Table 1). Data collection was carried out face-to-face in the clinic by the bilingual research assistant for self-reported questionnaires in most studies, except one study that utilised an online platform to collect the survey data [60]. The proportion of ethnic minority participants that make up the samples of the included studies varied. This included studies that solely focused on ethnic minority populations [60–62], and studies with 50% or more of participants from ethnic minority populations [59, 63–65]. The included seven studies adjusted for potential confounders, which included age [59, 61–65], gender [59–65], education [60, 62, 63, 65], income [61–63], health status [63], health insurance status [61, 64], years lived with T2DM [59, 60], self-efficacy [60], insulin use [59, 64], number of medications [59, 64], number of health conditions, and race/ethnicity [59, 63–65].

**Table 1:** Study characteristics and participant characteristics

Author-Year, Country	Study setting	Study design, Data collection Language	Sample- Number (n), Age years Mean (SD)	Race/Ethnicity	Years Diagnosed with Type 2 Diabetes Mellitus Mean (SD)	Income (USD)	Insurance
Ajuwon and Insel 2022 [60] Canada	Virtual through social media	Cross sectional study English	*N* = 53 Age—49.35 years (10.33)	African American (100%)	7.38 years (6.33)	≤$20,000 – 35.4% > $20,000 – 12.5%	Not known
White et al. 2013 [61] United States	Adult community based academic Internal Medicine clinic and two federally qualified health centres (FQHC) in Nashville, Tennessee	Cross sectional study Spanish	*N* = 149 Age—50 years (SD not reported)	Mexican (*n* = 116) (77.9%) Other Hispanics (*n* = 33) (22.1%)	5 years (SD not reported)	≤$10,000 -43.6% $10,000–$19,999 -37.6% >$20,000– 13.4%	Private – 28(18.8%) Uninsured – 121 (81.2%)
Garcia et al. 2019 [62] United States	A federally qualified health centre in San Diego County California	Cross sectional study (baseline data from RCT) English or Spanish	*N* = 279 Age—< 65 years—83.5% (233) > _65 years—16.5% (46) (SD not reported)	Hispanic/Latino of Mexican heritage (100%)	Not known	≤$20,000—65.2% > $20,000—34.8%	Private—5 (1.8%) Public—201 (72%) Uninsured—73 (26.2%)
Bains and Edege 2011 [63] United States	Internal Medicine Clinic at the Medical University of South Carolina, Charleston, South Carolina	Cross sectional study English	*N* = 125 Age—< 65 years- 63 (50.7%) 65 + years- 62 (49.3%) (SD not reported)	African American (71.4%) White (28.6%)	Not known	≤ $15,000—64.2%> $15,000—35.8%	Not Known
Fan et al. 2016 [64] United States	Barnes-Jewish Centre for Outpatient Health primary care clinic in St. Louis, Missouri	Cross sectional study English	*N* = 208 Age – 53 years (10.9)	Caucasian (26.4%) African American (73.6%)	Not Known	≤$20,000—78% > $20,000 – 22%	Private—22 (10.7%) Public—143 (69.4%) Uninsured—41 (19.9%)
Thurston et al. 2015 [65] United States	Three clinical sites for underserved patients in Athens and the metro-Atlanta communities in Georgia	Cross sectional study English	*N* = 192 Age – 55 years (10.3)	African American (76.6%) White (20.8%) Other (2.6%)	Not known	Low-income population- data not available	Not Known
Sarkar et al. 2006 [59] United States	Two primary care clinics at San Francisco General Hospital	Cross sectional study English or Spanish	*N* = 408 Age—58.1 years (11.4)	Asian/Pacific Islander (18%) African American (25%) Hispanic (40%) White/non-Hispanic (12%) Native American (0.5%) Multiethnic (1.5%) Other (3%)	9.5 years (8.0)	≤$20,000 – 93% > $20,000 – 7%	Not Known

### Participant characteristics

The mean age of participants from the included studies ranged from 49.4 to 70.0 years. The ethnic minority backgrounds of participants in the included studies were predominantly Hispanic [46, 59, 61, 62] comprising about 42% of total participants across seven studies and African-American [59, 60, 63–65] comprising about 39% of total participants across seven studies, Asian/Pacific islanders and other ethnic minority groups comprising about 10%, and remaining 9% comprising of white/non-Hispanic population of total participants across seven studies. The proportion of female participants in the included studies varied from 50% to 72.5% and the mean number of years diagnosed with T2DM ranged from 5 to 9.5.

### Methodological quality

All retained studies used a cross-sectional design and have utilised validated tools to measure health literacy and medication adherence. From the included seven studies, five studies [59–62, 65] addressed seven out of eight items on the JBI Appraisal checklist, and two studies [63, 64] addressed all eight items on the critical appraisal checklist (Table 2) (Appendix 6 for detailed checklist). Two studies [61, 65] did not describe the study subjects and the setting in detail and two studies [59, 62] did not use valid and reliable tools to measure the outcome. Retained studies had identified and adjusted for different potential confounders, among which common confounders were variables such as age, gender, education, income, health insurance status, years of T2DM, race, number of medication, and number of illnesses that can have an impact on both exposure and outcome measures, except for one study [60] which did not state the strategies to deal with confounding factors.

**Table 2:** Data collection tools utilised and results on the associations between health literacy and medication adherence

Author Year	Health literacy Instrument Value Mean SD	Medication adherence Instrument Value Mean SD	Association value	Statistical adjustment	Methodological Quality and Overall Appraisal (Appendix 6)
Ajuwon and Insel 2022 [60]	Brief Health Literacy Screening Tool	Simplified Medication Adherence Questionnaire	Health literacy was positively associated with medication adherence (*r* = 0.487, *p* = .001) in African American population	Adjusted for covariates gender, level of education, years of T2DM, perceived self-efficacy	Addressed 7 out of 8 items
White et al. 2013 [61]	Short Test of Functional Health Literacy in Adults (S-TOFHLA) Not Known	Summary of Diabetes Self-Care Activities (SDSCA) Not Known	Health literacy was not significantly associated with medication adherence AOR = 1.6; 95% CI: 0.9, 3.0, *p* = 0.11	Adjusted for covariates- age, gender, income, insurance status	Addressed 7 out of 8 items
Garcia et al. 2019 [62]	Newest Vital Sign (NVS) 1 item Adequate HL- 25 (9%) Limited HL—254 (91.0)	Proportion of Days Covered (PDC) PDC—0.20 (0.42%) Low MA- 201 (72%) Medium MA- 65 (23.2%) High MA—13 (4.7%)	Health literacy was not significantly associated with medication adherence, before adjusting for covariates in bivariate analysis (*P* = .35) and after adjusting covariates in multivariate analysis β = 0.16 (95% CI, 0.01–2.03)	Adjusted for covariates- age, gender, race, education, employment status, income	Addressed 7 out of 8 items
Bains and Edege 2011 [63]	Revised Rapid Estimate of Adult Literacy in Medicine (REALM-R) 6.1 + _ 0.3	Morisky medication adherence score- 4 item 0.9 (0.1)	Health literacy was not significantly associated with medication adherence, before adjusting for covariates in bivariate analysis and after adjusting covariates in multivariate analysis Spearman correlation Coefficient = 0.025, *P* = 0.794 After adjusting for covariates = -0.17 95% CI (-0.09 to 0.05)	Adjusted for covariates- Age, sex, Race, Education, Income, Health status	Addressed all 8 items
Fan et al. 2016 [64]	Brief Health Literacy Screen (BHLS)- 3 single item literacy screener Adequate health literacy -76 (36.5) Limited health literacy -132 (63.5)	Morisky Medication Adherence Scale (MMAS-4) Unintentional nonadherence (*n* = 208) 115 (55.3) Intentional nonadherence (*n* = 208) 83 (39.9)	Health literacy was positively associated with medication adherence in unadjusted bivariate analysis (β = 0.39, SE = 0.19, P = 0.037) Health literacy was not significantly associated with medication adherence after adjusting for covariates (β = 0.33, *P* = 0.22)	Adjusted for covariates—age, gender, race, insurance, diagnosis of depression, and medication regimen complexity	Addressed all 8 items
Thurston et al. 2015 [65]	The short-form Test of Functional Health Literacy in Adults (s-TOFHLA) 36-item timed reading comprehension test 32.8% had limited health literacy, 25.5(10)	The Morisky eight-item Medication Adherence Scale (MMAS-8) 58.9% had low adherence MMAS-8- 5.5 (1.8)	Health literacy was not significantly associated with medication adherence	Adjusted for covariates—age, gender, race, education, insulin use	Addressed 7 out of 8 items
Sarkar et al. 2006 [59]	The short version of the Test of Functional Health Literacy in Adults (s-TOHFLA)- Spanish or English version 0–16- inadequate health literacy—n (%)—156 (38.25) 17–22- marginal health literacy—54 (13.25) 23–36- adequate health literacy—198—(48.5)	The summary of Self-care activities questionnaire—how many diabetes pills patients have missed in last 7 days	Health literacy was not significantly associated with medication adherence	Adjusted for covariates -Age, gender, diabetes years, medication regimen, number of illnesses	Addressed 7 out of 8 items

### Health literacy measure

The tools used to measure health literacy among the included studies varied, including the 4-item Brief Health Literacy Screening (BHLS) Tool [60], the 3-item Brief Health Literacy Screen (BHLS) [64], a single-item literacy screener [64], the Rapid Estimate of Adult Literacy in Medicine Revised (REALM-R) [63], the short-form Test of Functional Health Literacy in Adults (s-TOFHLA) [59, 61, 65], and the single item Newest Vital Sign (NVS) [62].

### Medication adherence measure

In the included studies, medication adherence was measured using self-reported measures and prescription refill record. These included the 8-item Morisky Medication Adherence Scale (MMAS-8) [65], 4-item Morisky Medication Adherence Scale (MMAS-4) [63, 64], the Simplified Medication Adherence Questionnaire (SMAQ) [60], the medication engagement subscale of the Summary of Diabetes Self-Care Activities questionnaire (SDSCA) [59, 61] and Proportion of Days Covered (PDC) [62].

### Association between health literacy and medication adherence

Among the seven included studies, three studies solely targeted ethnic minority populations [60–62] and in the remaining four studies, at least 50% of participants identified as being from an ethnic minority background [59, 63–65] (Table 2).

### Studies solely focused on ethnic minority populations

Of the seven included studies, only three studies targeted ethnic minority populations. One study targeting participants from an African American background observed a significant association between health literacy level and medication adherence (*r* = 0.49, *p* = 0.001) [60]. Two studies that targeted people from Hispanic backgrounds did not find any association between health literacy level and medication adherence even after adjusting for covariates in the analysis [61, 62].

### Studies with 50% or more participants from an ethnic minority background

Among four studies with 50% or more participants from ethnic minority backgrounds, three studies observed no significant association between health literacy and medication adherence even after adusting for race as a covariate [59, 63, 65]. A study by Fan et al. [64] observed that health literacy was positively associated with medication adherence in the unadjusted bivariate analysis (β = 0.39, SE = 0.19, *P* = 0.037), but health literacy was not significantly associated with medication adherence after adjusting for covariates (β = 0.33, *P* = 0.22).

## Discussion

The objective of this systematic review was to examine the association between health literacy and medication adherence in individuals from ethnic minority backgrounds who have T2DM. This review highlights critical knowledge gaps in the existing literature, and methodological weaknesses of existing studies. It also highlights the unique challenges faced by ethnic minority groups such as cultural and linguistic barriers. By identifying the areas of insufficient evidence, this review highlights the critical need for further investigation targeting specific populations. Among retained studies, only one study observed a significant association between health literacy level and medication adherence among people from ethnic minority backgrounds, which solely targeted African American population [60]. Most of the studies (*n* = 6) were conducted in the United States and in most studies, participating ethnic minority groups were predominantly from African American and Hispanic backgrounds. The methodological quality of the studies ranged from good to fair, with most studies adjusting for socio-demographic variables to minimise the risk of bias due to confounders. The most common covariates being adjusted in all included studies were age, gender, educational level, income, years of T2DM, self-efficacy, number of medications, number of health conditions, and race/ethnicity.

Findings across studies included in this systematic review were inconsistent, which could be attributed to several factors. One of the key factors leading to inconsistency in the results is the use of different assessment tools to measure health literacy and medication adherence in people from different ethnicities living with T2DM. Some health literacy measures used in the included studies were self-reported, perception-based (subjective), and some were performance-based (objective) health literacy measures [66]. The included studies assessed different domains of health literacy such as numeracy, information seeking, pronunciation, comprehension, and general literacy. Combining both types of measures can give more accurate results when investigating health literacy and health outcomes rather than using only one type [67].

Moreover, all included studies measured general health literacy using health literacy tools (Appendix 4) rather than diabetes-specific health literacy. Two recent scoping reviews highlighted the diversity of instruments used to assess health literacy in patients with T2DM and observed that these instruments are validated in non-ethnic minority populations only, which are not recommended to be used in ethnic minority populations such as Hispanic and African Americans [68, 69]. Nonetheless, it is pleasing to note that newer health literacy assessment instruments specific to T2DM are being developed and validated worldwide [70, 71]. In terms of measurement of medication adherence, all included studies utilised different assessment tools (Appendix 5). Although all studies used validated instruments to assess medication adherence, one study [58] measured medication adherence by calculating the proportion of days covered for medication. It is noteworthy that instruments utilised by researchers in the included studies of this systematic review measured varying domains such as medication adherence, adherence to self-care activities including diet, physical activity, medication, and medication refill history. Such differences among instruments may lead to varying levels of sensitivity and specificity in measuring medication adherence constructs and therefore the lack of standardisation may lead to differences in the way health literacy and medication adherence were measured across studies. In the studies that did not exclusively focus on participants from ethnic minority backgrounds, the tools were not adapted for individuals who were non-English speakers or not predominantly English- speaking. The lack of culturally or linguistically appropriate tools may have contributed to differing findings between ethnic minority groups and others. Therefore, this makes it difficult to undertake a meta-analysis to pool the evidence from included studies.

The cross-sectional design employed in all the included studies is an another common factor that might have contributed to inconsistency in the results, limiting the ability to draw causal inferences from the findings [59]. The findings of this systematic review are consistent with another systematic review by Chima et al. [5]; although their review findings were not specifically focused on examining differences in the association between health literacy and medication adherence among ethnic minority population groups.

Across the seven studies, there were a variety of ethnic minority groups included, and only one study, which involved African Americans, reported an association between health literacy and medication adherence with ethnicity, however, cultural, and linguistic factors were not consistently identified as variables in any studies. Most studies collected data in the English language, with bilingual research staff or interpreters assisting participants with low English proficiency. However, only two studies [59, 62] translated the questionnaire from English to Spanish, and provided the option to the participant to respond in their preferred language. Tools used in the studies lacked cultural and linguistic sensitivity for non-English-speaking populations, a process called cross-cultural adaptation, which involves translating and culturally adapting the tool to ensure relevance in new settings [72]. This meticulous approach guarantees the reliability and validity of the instruments when used in diverse cultural and linguistic contexts [72]. People with low health literacy face challenges in understanding medication labels, dosage instructions and the importance of treatment regimens due to language barriers, low education levels, and acculturation levels in the host countries [73]. The language used in a questionnaire is crucial because, if it is not appropriate for a specific culture, the responses may not accurately reflect an individual’s health literacy and medication adherence [74].

Similar to language barriers, cultural beliefs are also an important factor behind shaping diabetes self-management behaviours, such as medication adherence, physical activity, and diet in ethnic minority populations [75]. The included studies did not focus on cultural beliefs and traditions that strongly influence illness perceptions, adherence to treatment regimens, and willingness to adhere to medications. The studies included a diverse range of ethnic minority populations, who may have different beliefs and practices which may explain the conflicting results. There can be multiple explanations behind non-adherence or low adherence to treatment regimens in people from different ethnicities, including the preference for complementary medicine and traditional remedies over allopathic medicine [76]. Some cultural beliefs support self-care activities that adjunct the therapeutic treatment, on the other hand, some may not support the utilisation of allopathic medicine. Socio-economic disparities intersect with health literacy and medication adherence in people living with T2DM, creating a complex web of interconnected factors that significantly impact the management of T2DM in ethnic minority populations.

Although there is some evidence of the association between health literacy and medication adherence, this was inconclusive primarily attributed to variations in the assessment methods for health literacy and medication adherence, as well as the diverse range of ethnic minority groups included across the studies. Addressing cultural beliefs, language barriers, and socio-economic disparities is critical for improving medication adherence and diabetes self-management in ethnic minority populations. There is a need for studies focusing on specific culturally and linguistically diverse (CALD) groups rather than broad categorisations of ethnicity/race. There was one study with 35% African American participants [77] which was excluded from the review due to the low percentage of the target population for this review. In this study, after adjusting for covariates in their multivariate analysis, they reported an association between African American ethnicity and poor medication adherence, but not between health literacy and medication adherence in the African American population [77]. This explains why it is necessary to specifically recruit CALD communities that are not entangled with a predominately white population, and therefore can provide accurate results.

It is critical to address the disparities in cultural and linguistic considerations within healthcare research. Economic disparities and limited access to resources exacerbate challenges faced by individuals with lower health literacy, resulting in disparities in understanding and adhering to medication regimens. This complex scenario underscores the inequities in diabetes management, emphasising the need for a more equitable approach to T2DM care within ethnic minority communities. A comprehensive approach, incorporating cross-cultural adaptation and a nuanced understanding of cultural beliefs, is crucial to ensuring that health interventions are accessible, relevant, and effective across diverse populations. Achieving equity in healthcare requires acknowledging and dismantling barriers, whether they be language-related, cultural, or socioeconomic, to ensure that all individuals, have equal access and understanding of vital health information and resources. Further research should investigate these factors insightfully to co-design, implement, and evaluate interventions to improve medication adherence and health literacy among ethnic minority adults living with T2DM. Also, future research should consider the lifestyle or self-management interventions, because they also have significant impact in diabetes management. In addition, population segmentation can play a crucial role in identifying subgroups of ethnic minorities with varying health literacy and medication adherence [78]. Further studies are needed to identify the optimal segmentation frameworks that consider factors such as cultural differences, socioeconomic status, and health literacy levels to ensure effective and equitable healthcare delivery.

### Implications for policy

The findings from this study highlights the gap in existing literature, which necessitates comprehensive and culturally informed strategies to address this gap. The existing literature does not incorporate cultural and linguistic factors in the research and therefore, future research should focus on investigating the relationship between health literacy and medication adherence among ethnic minority populations with a specific focus on cultural and linguistic barriers and utilise validated tools for specific populations. It is important to understand these factors, as this can assist policymakers and health professionals in designing targeted interventions and providing appropriate support and practical advice to ethnic minority people living with T2DM. Advocating for the inclusion of diverse populations in research studies can provide policymakers with a more accurate representation of their experiences and needs.

Support from health professionals can have an impact on the health outcomes of those from ethnic minority backgrounds, and it requires health professionals to employ strategies to ensure patients understand the disease process, prevention, and management. This includes using plain language [79], simple communication [79], visual aids [80, 81], and the teach-back method [82], where clinicians can verify patient understanding and so can improve health literacy and their health outcomes [83]. Staff training in health literacy and culturally safe healthcare practices is crucial [84]. Additionally, availability of easily readable written materials and education about health conditions can help in improving health literacy [85–87]. This has implications for clinical practice and policymaking, thus policymakers should support the modification of health services environments and the development of policies or frameworks that promote these practices to improve health outcomes for ethnic minority populations with T2DM.

### Strengths and limitations

To the best of our knowledge, this is the first systematic review to explore the association of health literacy and medication adherence in ethnic minority adults with T2DM. An extensive search was conducted in five electronic databases, and a thorough search strategy was developed in consultation with a health science librarian to ensure the inclusion of a wide range of relevant evidence and to reduce the risk of selection bias. The JBI critical appraisal tool was utilised to assess the methodological quality of all included studies, which enhances the credibility and rigour of the review’s findings. Existing systematic reviews were reviewed and identified extensive knowledge gaps in the research focusing on ethnic minority population with T2DM.

The cross-sectional design of all the included studies limits the ability to establish causality. A meta-analysis was not possible as the included studies assessed health literacy and medication adherence using different instruments, varying sample sizes, varying percentages of ethnic minority populations, age groups, and the results were not disaggregated by ethnic categories. The heterogeneity in sample characteristics made it difficult to interpret and combine effect sizes across studies. Another limitation of this review is the exclusion of grey literature, which may contribute to publication bias. Only studies published in the English language were included in this review and therefore it is possible that studies in other languages were not included in the review findings. Another limitation is that a diverse range of ethnic minority populations were included in the review, with the most represented ethnic minority groups from African American and Hispanic backgrounds.

## Conclusion

Evidence on the association between health literacy and medication adherence in ethnic minority adults with T2DM is weak and inconsistent. All study designs were cross-sectional; therefore, any causal inferences were not possible. To understand this association more clearly in ethnic minority populations and the impact of cultural and linguistic factors, well-designed studies are required.

## Supplementary Information

Additional file 1: Appendix 1. Existing Systematic reviews. Appendix 2. Search terms. Appendix 3. Search strategy for all 5 Databases. Appendix 4. Health literacy measurement tools. Appendix 5. Medication Adherence Measurement tools. Appendix 6. Assessment of methodological quality of the retained studies. Appendix 7. Reasons for exclusion of studies. Appendix 8(a). PRISMA Checklist. Appendix 8(b). PRISMA Abstract Checklist. Appendix 9. Data Extraction Form.

## Data Availability

No datasets were generated or analysed during the current study.
