# Supplementary material for: Health literacy and medication adherence in adults from ethnic minority backgrounds with Type 2 Diabetes Mellitus: a systematic review
Source: BMC Public Health. 2025 Jan 20;25:222. doi: 10.1186/s12889-024-20734-z (PMC11745004; doi:10.1186/s12889-024-20734-z)
Supplement: Supplementary file 1 — Additional file 1: Appendix 1. Existing Systematic reviews. Appendix 2. Search terms. Appendix 3. Search strategy for all 5 Databases. Appendix 4. Health literacy measurement tools. Appendix 5. Medication Adherence Measurement tools. Appendix 6. Assessment of methodological quality of the retained studies. Appendix 7. Reasons for exclusion of studies. Appendix 8(a). PRISMA Checklist. Appendix 8(b). PRISMA Abstract Checklist. Appendix 9. Data Extraction Form. [file 12889_2024_20734_MOESM1_ESM.docx]

**APPENDICES**

Appendix 1: Existing Systematic reviews

| **No** | **Reference** | **Title of Article** | **Chronic Disease** | **Search End Date** | **Research Question** | **Included Study Designs** | **Databases Searched** | **Association between HL and MA** | **Statistical Significance of association** | **Linguistic and Cultural barriers or migrant population considered** | **Participants Age Range** | **Countries of study included in SR** | **Intervention Based or Non-Intervention Study** | **AMSTAR 2 rating** |
| --- | --- | --- | --- | --- | --- | --- | --- | --- | --- | --- | --- | --- | --- | --- |
| 1 | Chima et.al. 2020 | Impact of Health Literacy on Medication Engagement Among Adults with Diabetes in the United States: A Systematic Review | Diabetes Mellitus | Apr-20 | Impact of Health Literacy on Medication Engagement Among Adults with Diabetes in the United States | Cross Sectional Designs-9, Quasi-Experimental Studies-2 | PubMed, Ovid Medline, CINAHL, Embase, PsycInfo, and Scopus | Yes | Statistically significant But Weak | No, none of the studies included non-fluent English speakers | ≥18-year-old | USA | Considered both- Intervention and Non-intervention studies | low |
| 2 | Tan et.al. 2019 | A systematic review and meta‐analysis on the effectiveness of education on medication adherence for patients with hypertension, hyperlipidaemia, and diabetes | Diabetes Mellitus, Hypertension, Hyperlipidaemia | Dec-16 | Effectiveness of education on medication adherence for patients with hypertension, hyperlipidaemia, and diabetes | RCTs | PubMed, Embase, CINAHL, Cochrane Central Register of Controlled Trials (CENTRAL), Science direct, Scopus and Web of Science | No | correlation between Medication adherence and intervention shown, but no between HL and MA | No, not focusing on minor-ethnic group | ≥18-year-old | Not Specified | Intervention Based | Critically low |
| 3 | Su Hyun 2016 | [Health-Literacy-Sensitive Diabetes Self-Management Interventions: A Systematic Review and Meta-Analysis](https://west-sydney-primo.hosted.exlibrisgroup.com/primo-explore/fulldisplay?docid=TN_cdi_gale_infotracacademiconefile_A461101957&context=PC&vid=UWS-ALMA&lang=en_US&search_scope=default_scope&adaptor=primo_central_multiple_fe&tab=default_tab&query=any%2Ccontains%2CHealth%20literacy%20and%20health%20outcomes%20in%20diabetes%3A%20a%20systematic%20review.&offset=0) | Diabetes Mellitus | Jan-15 | health-literacy-sensitive diabetes management interventions to improve health outcomes | RCTs | PubMed, CINAHL, and EMBASE | No | Interventions were effective for improving glycaemic control, but not focused on medication adherence | Strategies for intervention considered language and culture | Not specified | Not Specified | Intervention Based | Critically low |
| 4 | Loke et.al.2012 | Review links between health literacy and cardiovascular/diabetes medication adherence. | Diabetes Mellitus, Cardiac disease | Feb-12 | Systematic Review of Consistency Between Adherence to Cardiovascular or Diabetes Medication and Health Literacy in Older Adults | Cross-sectional study, Prospective cohort study, Retrospective cohort study, Intervention Trial | Embase and Medline | No | Does not show any association | No, not focusing on minor-ethnic group | ≥50-year-old | USA | Non-Intervention Studies | Critically low |
| 5 | Al Sayah et. Al. 2012 | Health Literacy and Health Outcomes in Diabetes: A Systematic Review | Diabetes Mellitus | Mar-12 | Improve understanding of relationship between health literacy or numeracy and health outcomes in diabetes. | Cross-sectional longitudinal | CINAHL, Embase, ERIC, Medline, psycINFO, and SCOPUS | No | weak evidence on correlation between health literacy and diabetes related outcome, but no association between HL and MA | No, not focusing on minor-ethnic group | Not specified | USA | Non-Intervention Studies | Critically low |
| 6 | Sheridan, 2011 | Interventions for individuals with low health literacy: a systematic review. | Diabetes Mellitus | May-10 | Effective interventions for people with low health literacy to improve use of health care services, improve health outcomes, affect the costs of care, and reduce disparities | RCT, cRCT, NRCT and quasi-experimental studies | MEDLINE, CINAHL, PsycINFO, Educational Resources Information Center (ERIC), and the Cochrane Library | No | Evidence on effectiveness of intervention on self-management but no association shown | Considered health outcome in racial, ethnic, cultural, or age groups in research question but result does not show any focus | Not specified | USA, Germany, New Zealand | Intervention Based | Critically low |
| 7 | Fransen et. al. 2012 | Diabetes self-management in patients with low health literacy: ordering findings from literature in a health literacy framework. | Diabetes Mellitus | Jul-11 | Explore possible associations between health literacy, diabetes self-management, and possible mediators. | Cross-sectional survey, Prospective observational study, intervention study | PubMed | No | weak and mixed association between HL and Diabetes self-management/medication adherence | No, not focusing on minor-ethnic group | ≥50-year-old | USA-10, UK-1 | Considered both- Intervention and Non-intervention studies | Critically low |
| 8 | Van Scoyoc, 2010 | Interventions to improve diabetes outcomes for people with low literacy and numeracy: a systematic literature review. | Diabetes Mellitus | Jan-10 | Explore interventions which improve outcomes among patients with diabetes and low literacy | RCT, Pre-post design study | Medline | No | Association between low literacy and diabetes outcome but not health literacy and medication adherence/diabetes management | No, not focusing on minor-ethnic group | Not specified | Not Specified | Intervention Based | Critically low |
| 9 | Schaefer, 2008 | Integrated review of health literacy interventions | Diabetes Mellitus, HIV | Dec-06 | Explore health literacy interventions | Not specified | CINAHL and MEDLINE | No | Weak evidence | No, not focusing on minor-ethnic group | ≥18-year-old | Not Specified | Intervention Based | Critically low |
| 10 | Wali et.al. 2016 | A systematic review of interventions to improve medication information for low health literate populations | Diabetes Mellitus, Hypertension, Heart disease, rheumatic disease, asthma, hyperlipidaemia | Mar-15 | A systematic review of interventions to improve medication information for low health literate populations | RCT, NRCT, UCT | PubMed, Embase, IPA, Web of Science, Cochrane Library, CINAHL, PsycINFO, and Scopus | Yes | Statistically significant | No (Ethnic minority considered as demographic characteristic affecting Health literacy but not focused) | >50 years | USA (Max number of studies), UK, Canada, Europe, South Africa, Australia, Iran, Japan, Turkey | Intervention Based | low |
| 11 | Miller 2016 | Health literacy and adherence to medical treatment in chronic and acute illness: A meta-analysis | Diabetes Mellitus, Cardiac, Renal, Pulmonary, GI, Blood, respiratory diseases, Arthritis | Dec-12 | Explore relationship between patient health literacy and treatment adherence, interventions to improve health literacy and medication adherence | Co-relational studies, Intervention studies | Psychinfo, PubMed | Yes | Positive and statistically significant correlation | No (Ethnicity considered as a factor affecting HL) (Compared Caucasian vs Not-Caucasians), Minor-ethnic group were considered | Not specified | USA and other countries (Name not specified) | Intervention Based | Critically low |
| 12 | Keller et.al. 2008 | Impact of health literacy on health outcomes in ambulatory care patients: a systematic review. | Diabetes Mellitus, Hypertension, Coronary Heart disease, rheumatic disease, asthma, glaucoma, hyperlipidaemia | Apr-08 | Consider how low health literacy relates to disease state control or medication adherence. | Cross Sectional study, prospective study | Cochrane, MEDLINE, CINAHL, EMBASE, Education Resources Information Center, PsycINFO, IPA and Iowa Drug In- formation Service. | Yes | Positive but weak correlation | No (Mentioned Ethnicity and English as second language are at high risk of low HL but not considered as a factor and so controlled by author in the study) | ≥65-year-old | Not Specified | Non-Intervention Studies | Critically low |
| 13 | Zhang et.al. 2014 | Impact of Health Literacy on Medication Adherence: A Systematic Review and Meta-analysis | Diabetes Mellitus, Hypertension, Coronary Heart disease, rheumatic disease, asthma, glaucoma, hyperlipidaemia, IV/AIDS | May-13 | The relationship between health literacy and medication adherence through meta-analysis | Cross Sectional study, prospective study, interview, observational study | MEDLINE, IPA, PsycINFO, CINAHL, and Web of Knowledge | yes | Statistically significant but weak association | No | Not specified | USA, Canada, Europe, New Zealand, Australia | Considered both- Intervention and Non-intervention studies | Critically low |

**Appendix 2. Search terms of the tailored search strategy for the systematic review**

|  | Search Terms |
| --- | --- |
| Population | Diabetes mellitus OR Diabetes Mellitus, Type 2 OR Diabet* OR mellitus OR T2DM |
| Exposure of Interest | Health litera* OR health behaviour OR health belief OR health practice OR health knowledge OR health status disparities |
| Outcome | Medication adherence OR drug adherence OR treatment adherence OR medication non- adherence OR treatment non-adherence OR patient complian* OR medication complian* OR treatment complian* OR medication non- complian* OR treatment non-complian* OR medication engagement OR medication management OR persistence OR treatment refusal OR therapeutics OR patient education as topic |

**Appendix 3. Search strategy for all 5 Databases**

1. **Search strategy for MEDLINE (OVID) (23rd Jan 2024)**

| # | Searches | Result |
| --- | --- | --- |
| 1 | Health Literacy/ or Health Behaviour/ or Health Belief/ or Health Status Disparities/ | 29404 |
| 2 | Health adj2 (liter* or practice* or Attitude* or Knowledge* or behavio?r* or belie*)).ti,ab. | 119372 |
| 3 | 1 or 2 | 140492 |
| 4 | Patient compliance/ or Medication adherence/ or Therapeutics/ or Medication engagement/ or Medication management/ or Patient Education/ | 174181 |
| 5 | ((Medicat* or drug* or treatment* or therap*) adj2 (adher* or non-adher* or nonadher* or Complian* or non- complian* or noncomplian* or engag* or nonengagement or persist* or refus* or manag*)). ti, ab. | 133014 |
| 6 | 4 or 5 | 282263 |
| 7 | Diabetes mellitus/ or Diabetes Mellitus, Type 2/ | 314024 |
| 8 | (Diabet* or mellitus or DMT2). ti, ab. | 791429 |
| 9 | 7 or 8 | 828156 |
| 10 | 3 and 6 and 9 | 1166 |

1. **Search strategy for Embase (OVID) (23^rd^ Jan 2024)**

| # | Searches | Results |
| --- | --- | --- |
| 1 | Health Literacy/ or Health Behaviour/ or Health Belief/ or Health Status Disparities/ | 145,756 |
| 2 | (Health adj2 (liter* or practice* or Attitude* or Knowledge* or behavio?r* or belie*)).ti,ab. | 142,042 |
| 3 | 1 or 2 | 238,318 |
| 4 | Patient compliance/ or Medication adherence/ or Therapeutics/ or Medication engagement/ or Medication management/ or Patient Education/ | 1,706,357 |
| 5 | ((Medicat* or drug* or treatment* or therap*) adj2 (adher* or non-adher* or nonadher* or Complian* or non- complian* or noncomplian* or engag* or nonengagement or persist* or refus* or manag*)).ti,ab. | 212,518 |
| 6 | 4 or 5 | 1,848,270 |
| 7 | Diabetes mellitus/ or Diabetes Mellitus, Type 2/ | 897,442 |
| 8 | (Diabet* or mellitus or DMT2).ti,ab. | 1,251,404 |
| 9 | 7 or 8 | 1,461,356 |
| 10 | 3 and 6 and 9 | 2,823 |

1. **Search strategy for CINAHL (EBSCohost) (23^rd^ Jan 2024)**

| # | Searches | Results |
| --- | --- | --- |
| S10 | S3 AND S6 AND S9 | 813 |
| S9 | S7 OR S8 | 242,575 |
| S8 | TI (Diabet* or mellitus or DMT2) OR AB (Diabet* or mellitus or DMT2) | 221,965 |
| S7 | (MH "Diabetes mellitus") OR (MH "Diabetes Mellitus, Type 2") | 121,440 |
| S6 | S4 OR S5 | 97,384 |
| S5 | TI ((Medicat* or drug* or treatment* or therap*) N2 (adher* or non-adher* or nonadher* or Complian* or non- complian* or noncomplian* or engag* or nonengagement or persist* or refus* or manag*)) OR AB ((Medicat* or drug* or treatment* or therap*) N2 (adher* or non-adher* or nonadher* or Complian* or non- complian* or noncomplian* or engag* or nonengagement or persist* or refus* or manag*)) | 66,600 |
| S4 | (MH "Patient compliance") OR (MH "Medication adherence") OR (MH "Therapeutics") OR (MH "Medication engagement") OR (MH "Medication management") OR (MH "Patient Education as topic") | 37,441 |
| S3 | S1 OR S2 | 125,283 |
| S2 | (MH "Health Status Disparities") OR (MH "Health Beliefs") OR (MH "Health Behavior") | 72,723 |
| S1 | TI ((Health) N2 (liter* or practice* or Attitude* or Knowledge* or behavio?r* or belie*)) OR AB ((Health) N2 (liter* or practice* or Attitude* or Knowledge* or behavio?r* or belie*)) | 62,556 |

1. **Search strategy for PsychInfo (EBSCOhost) (23^rd^ Jan 2024)**

| # | Searches | Results |
| --- | --- | --- |
| S10 | S3 AND S6 AND S9 | 491 |
| S9 | S7 OR S8 | 37,939 |
| S8 | TI (Diabet* or mellitus or DMT2) OR AB (Diabet* or mellitus or DMT2) | 37,311 |
| S7 | MA Diabetes mellitus OR MA Diabetes Mellitus, Type 2 | 10,896 |
| S6 | S4 OR S5 | 67,314 |
| S5 | TI ((Medicat* or drug* or treatment* or therap*) N2 (adher* or non-adher* or nonadher* or Complian* or non- complian* or noncomplian* or engag* or nonengagement or persist* or refus* or manag*)) OR AB ((Medicat* or drug* or treatment* or therap*) N2 (adher* or non-adher* or nonadher* or Complian* or non- complian* or noncomplian* or engag* or nonengagement or persist* or refus* or manag*)) | 46,761 |
| S4 | MA Patient compliance OR MA Medication adherence OR MA Therapeutics OR MA Medication engagement OR MA Medication management OR MA Patient Education as Topic | 27,321 |
| S3 | S1 OR S2 | 64,942 |
| S2 | MA Health literacy OR MA Health Status Disparities OR MA Health Beliefs OR MA Health Behavior | 22,312 |
| S1 | TI ((Health) N2 (liter* or practice* or Attitude* or Knowledge* or behavio?r* or belie*) OR AB ((Health) N2 (liter* or practice* or Attitude* or Knowledge* or behavio?r* or belie*) | 46,705 |

1. **Search strategy for Cochrane (23^rd^ Jan 2024)**

| # | Search Hits | Results |
| --- | --- | --- |
| #1 | ("Health Literacy" or "Health Behaviour" or "Health Belief" or "Health Status Disparities"):ti,ab,kw | 13,546 |
| #2 | (Health near/2 (liter* or practice* or Attitude* or Knowledge* or behavio?r* or belie*)):ti,ab,kw | 37,369 |
| #3 | #1 OR #2 | 37,594 |
| #4 | (("Patient compliance" or "Medication adherence" or "Therapeutics" or "Medication engagement" or "Medication management" or "Patient Education as topic")):ti,ab,kw | 44,308 |
| #5 | ((Medicat* or drug* or treatment* or therap*) near/2 (adher* or nonadher* or non-adher* or Complian* or non-complian* or noncomplian* or engag* or nonengagement or persist* or refus* or manag*)):ti,ab,kw | 40,562 |
| #6 | #4 OR #5 | 70,765 |
| #7 | ("Diabetes mellitus" or "Diabetes Mellitus, Type 2"):ti,ab,kw | 82,556 |
| #8 | (Diabet* or mellitus or DMT2):ti,ab,kw | 119,856 |
| #9 | #7 OR #8 | 119,856 |
| #10 | #3 AND #6 AND #9 | 1,025 |

**Appendix 4. Health literacy measurement tools**

| Health Literacy Measurement Tool | Health literacy Score range |
| --- | --- |
| Rapid Estimate of Adult Literacy in Medicine (REALM) | Total score -number of correctly pronounced words |
| Revised Rapid Estimate of Adult Literacy in Medicine (REALM-R) | 0 to 8 (score 6 or less - poor health literacy) |
| Short Test of Functional Health Literacy in Adults (S-TOFHLA) | Score Range- 0-36, Scores of 0 - 16, 17 - 22, and >23 indicates inadequate, marginal, and adequate HL, respectively. |
| Brief Health Literacy Screen (BHLS) | Scored on 4-point scale- 12-point BHLS index. 0 to 2 - adequate health literacy,  3 to 12 - limited health literacy |
| 6-item Newest Vital Sign (NVS) | incorrect = 0, correct=1  score range - 0 to 6,  scores 4-6 = adequate health literacy,  scores 0-3= inadequate health literacy |
| Newest Vital Sign (NVS) 1 item | Responses coded as correct (0) (adequate health literacy) or incorrect (1) (limited health literacy) |

**Appendix 5. Medication Adherence Measurement tools**

| Medication Adherence Measurement Tool | Medication adherence Score range |
| --- | --- |
| Simplified Medication Adherence Questionnaire | range of 0-9, in which a higher score indicates a higher adherence |
| Morisky Medication Adherence  Scale (MMAS-4) | Yes or No, score range from 0-8, 6 or above- adherent, 0-5 low adherence |
| The Summary of Diabetes Self-Care Activities (SDSCA) questionnaire- | how many diabetes pills patients have missed in last 7 days, Total score- 0-7 days, number of days being adherent to anti-diabetic medicine |
| The Morisky eight-item Medication Adherence Scale (MMAS-8) | summation of the 8 items ranges from 0 to 8,  a score below 6 -low medication adherence,  6 to <8 implies moderate medication adherence,  8- High level of medication adherence |
| 11-item adherence to Refills and Medications Scale for Diabetes (ARMS-D) 7-item medication-taking subscale and a 4-item medication refill subscale | summed and range from 11 to 44  4-point Likert-type scale- score ranging from 11 to 44 Total sub-score 28 - High-adherent score below 28- low-adherent Total sub score 16 for refiling- better adherence |
| Proportion of Days Covered (PDC) | Sum of days covered divided by days monitored Score range- 0.00 to 1.00 optimal/high adherence (PDC >_ 0.80), medium adherence (>0.50‚ 0.79), and low adherence (<_0.50) |

**Appendix 6. Assessment of methodological quality of the retained studies**

| Study ID | 1. Were the criteria for inclusion in the sample clearly defined? | 2. Were the study subjects and the setting described in detail? | 3. Was the exposure measured in valid and reliable way? | 4. Were objective, standard criteria used for measurement of condition? | 5. Were confounding factors identified? | 6. Were strategies to deal with confounding factors stated? | 7. Were the outcomes measured in valid and reliable way? | 8. Was appropriate statistical analysis used? | Overall appraisal- Include, exclude or Seek further info |
| --- | --- | --- | --- | --- | --- | --- | --- | --- | --- |
| Ajuwon 2022 | 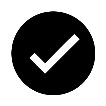 | 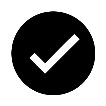 | 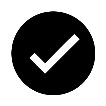 | 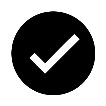 | 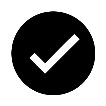 | 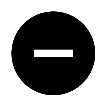 | 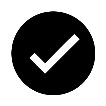 | 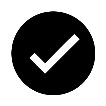 | Include |
| Sarkar 2006 | 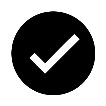 | 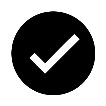 | 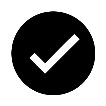 | 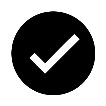 | 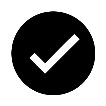 | 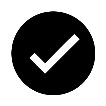 | 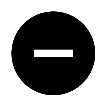 | 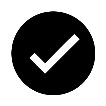 | Include |
| Bains 2011 | 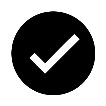 | 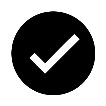 | 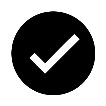 | 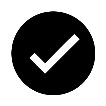 | 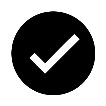 | 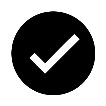 | 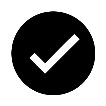 | 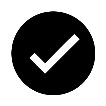 | Include |
| Thurston 2015 | 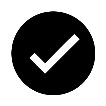 | 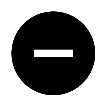 | 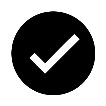 | 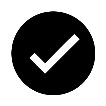 | 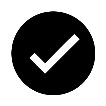 | 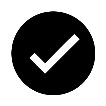 | 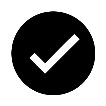 | 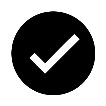 | Include |
| Fan 2016 | 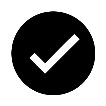 | 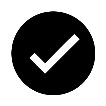 | 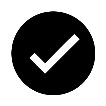 | 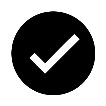 | 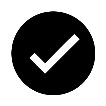 | 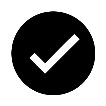 | 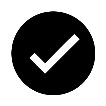 | 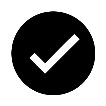 | Include |
| Garcia 2019 | 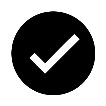 | 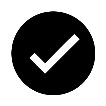 | 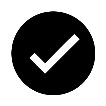 | 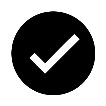 | 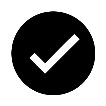 | 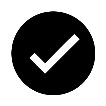 | 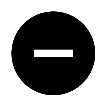 | 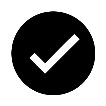 | Include |
| White 2013 | 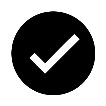 | 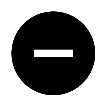 | 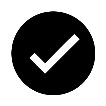 | 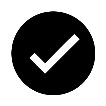 | 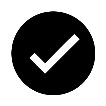 | 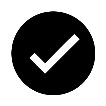 | 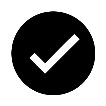 | 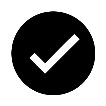 | Include |

**Appendix 7. Reasons for exclusion of studies**

| Author | Reason for exclusion |
| --- | --- |
| Elsous et al. 2017 (1) | Non-CALD study – Has not involved ethnic minority/CALD study participants |
| Zeng et al. 2014 (2) | Non-English study - Available in Chinese language |
| Lee et al. 2016 (3) | Non-CALD study – Has not involved ethnic minority/CALD study participants |
| Huang et al. 2017 (4) | Duplicate - Duplicate study of the include study in the review |
| Morris et al. 2006 (5) | Wrong comparator - Assessed health literacy, physiologic control, and diabetes complications among adults with diabetes |
| Marmouch et al. 2021 (6) | Non-CALD study – Has not involved ethnic minority/CALD study participants |
| Yeung et al. 2018 (7) | Wrong comparator - Assessed association between diabetes knowledge, self-care behaviours, medication adherence and depressive symptoms |
| Krzeminska et al. 2021 (8) | Wrong outcomes- Assessed the impact of self-care on adherence to treatment in patients with T2DM |
| Kassahun et al. 2016 (9) | Non-CALD study – Has not involved ethnic minority/CALD study participants |
| Bauer et al. 2013 (10) | Wrong outcomes- Assessed association between health literacy and antidepressant medication adherence, and non-CALD study |
| White et al. 2010 (11) | Duplicate - Duplicate study of the include study in the review |
| Todd et al. 2015 (12) | Non-CALD study – Has not involved ethnic minority/CALD study participants |
| Leemans et al. 2021 (13) | Non-English study - Available in French language |
| Shiyanbola et al. 2017 (14) | Non-CALD study – Has not involved ethnic minority/CALD study participants |
| Osborn et al. 2011 (15) | Duplicate - Duplicate study of the include study in the review |
| Chin et al. 2021 (16) | Wrong comparator - Assessed association between health literacy and memory for medication purposes |
| Ilhan et al. 2021(17) | Non-CALD study – Has not involved ethnic minority/CALD study participants, and Wrong comparator - Assessed association between health literacy and diabetes self-care |
| Thurston et al. 2013 (18) | Non-CALD study – Has not involved ethnic minority/CALD study participants |
| Kooshyar et al. 2014 (19) | Non-CALD study – Has not involved ethnic minority/CALD study participants |
| Hur et al. 2020 (20) | Non-CALD study – Has not involved ethnic minority/CALD study participants |
| Selvakumar et al. 2023 (21) | Non-CALD study – Has not involved ethnic minority/CALD study participants |
| Schillinger 2021 (22) | Wrong outcome – Validity study of health literacy measure |
| Olorunfemi 2023 (23) | Non-CALD study – Has not involved ethnic minority/CALD study participants |
| Crespo et al. 2020 (24) | Wrong outcome – Assessed correlation between cognitive function and health literacy, medication adherence was assessed but not analysed to find association with health literacy |
| Ueno et al. 2019 (25) | Non-CALD study – Has not involved ethnic minority/CALD study participants |
| Sharma et al. 2023 (26) | Non-CALD study – Has not involved ethnic minority/CALD study participants |
| Osborn et al. 2011 (27) | Duplicate - Duplicate study of the include study in the review |
| Huang and Shiyanbola 2021 (28) | Non-CALD study – Has not involved ethnic minority/CALD study participants |
| Radwan et al. 2018 (29) | Non-CALD study – Has not involved ethnic minority/CALD study participants |
| Rocha et al. 2019 (30) | Non-CALD study – Has not involved ethnic minority/CALD study participants |
| Tefera et al. 2020 (31) | Non-CALD study – Has not involved ethnic minority/CALD study participants |
| Hussain et al. 2020 (32) | Non-CALD study – Has not involved ethnic minority/CALD study participants |
| Ng and Park 2021 (33) | Non-CALD study – Has not involved ethnic minority/CALD study participants |
| Hagliroshan 2021 (34) | Non-English study - Available in Arabic language |
| Ozonuk and Yilmaz 2019 (35) | Non-English study - Available in Turkish language |
| Yeh et al. 2018 (36) | Non-CALD study – Has not involved ethnic minority/CALD study participants |
| Opsasnick et al. 2019 (37) | Wrong Comparator – Assessed association of medication adherence with limited literacy and English proficiency |
| Chu 2010 (38) | Non-CALD study – Has not involved ethnic minority/CALD study participants |
| Nandyala 2018 (39) | Non-CALD study – Has involved less than 50% participants from ethnic minority background |
| Huang 2020 (40) | Non-CALD study – Has involved less than 50% participants from ethnic minority background |
| Shiyanbola 2018 (41), Huang 2018 (42), Huang 2018 (43) (3 articles representing 1 unique study) | Non-CALD study – Has involved less than 50% participants from ethnic minority background |
| Osborn 2011 (44) | Non-CALD study – Has involved less than 50% participants from ethnic minority background |

**Appendix 8a). PRISMA Checklist**

| **Section and Topic** | **Item #** | **Checklist item** | **Location where item is reported** |
| --- | --- | --- | --- |
| **TITLE** | | |  |
| Title | 1 | Identify the report as a systematic review. | Page 1-2 |
| **ABSTRACT** | | |  |
| Abstract | 2 | See the PRISMA 2020 for Abstracts checklist. | Yes, See appendix 6(b) |
| **INTRODUCTION** | | |  |
| Rationale | 3 | Describe the rationale for the review in the context of existing knowledge. | Page 7-8 |
| Objectives | 4 | Provide an explicit statement of the objective(s) or question(s) the review addresses. | Page 08 |
| **METHODS** | | |  |
| Eligibility criteria | 5 | Specify the inclusion and exclusion criteria for the review and how studies were grouped for the syntheses. | Page 8-9 |
| Information sources | 6 | Specify all databases, registers, websites, organisations, reference lists and other sources searched or consulted to identify studies. Specify the date when each source was last searched or consulted. | Page 09 |
| Search strategy | 7 | Present the full search strategies for all databases, registers and websites, including any filters and limits used. | Page 09 |
| Selection process | 8 | Specify the methods used to decide whether a study met the inclusion criteria of the review, including how many reviewers screened each record and each report retrieved, whether they worked independently, and if applicable, details of automation tools used in the process. | Page 9-10 |
| Data collection process | 9 | Specify the methods used to collect data from reports, including how many reviewers collected data from each report, whether they worked independently, any processes for obtaining or confirming data from study investigators, and if applicable, details of automation tools used in the process. | Page 10 |
| Data items | 10a | List and define all outcomes for which data were sought. Specify whether all results that were compatible with each outcome domain in each study were sought (e.g. for all measures, time points, analyses), and if not, the methods used to decide which results to collect. | Page 8,10 |
|  | 10b | List and define all other variables for which data were sought (e.g. participant and intervention characteristics, funding sources). Describe any assumptions made about any missing or unclear information. | Page 10 |
| Study risk of bias assessment | 11 | Specify the methods used to assess risk of bias in the included studies, including details of the tool(s) used, how many reviewers assessed each study and whether they worked independently, and if applicable, details of automation tools used in the process. | Page 10-11 |
| Effect measures | 12 | Specify for each outcome the effect measure(s) (e.g. risk ratio, mean difference) used in the synthesis or presentation of results. | N/A |
| Synthesis methods | 13a | Describe the processes used to decide which studies were eligible for each synthesis (e.g. tabulating the study intervention characteristics and comparing against the planned groups for each synthesis (item #5)). | Page 10-11 |
|  | 13b | Describe any methods required to prepare the data for presentation or synthesis, such as handling of missing summary statistics, or data conversions. | N/A |
|  | 13c | Describe any methods used to tabulate or visually display results of individual studies and syntheses. | Page 10-11 |
|  | 13d | Describe any methods used to synthesize results and provide a rationale for the choice(s). If meta-analysis was performed, describe the model(s), method(s) to identify the presence and extent of statistical heterogeneity, and software package(s) used. | Page 10-11 |
|  | 13e | Describe any methods used to explore possible causes of heterogeneity among study results (e.g. subgroup analysis, meta-regression). | Page 12 |
|  | 13f | Describe any sensitivity analyses conducted to assess robustness of the synthesized results. | N/A |
| Reporting bias assessment | 14 | Describe any methods used to assess risk of bias due to missing results in a synthesis (arising from reporting biases). | N/A |
| Certainty assessment | 15 | Describe any methods used to assess certainty (or confidence) in the body of evidence for an outcome. | Page 11-12 |
| **RESULTS** | | |  |
| Study selection | 16a | Describe the results of the search and selection process, from the number of records identified in the search to the number of studies included in the review, ideally using a flow diagram. | Page 12 |
|  | 16b | Cite studies that might appear to meet the inclusion criteria, but which were excluded, and explain why they were excluded. | Page 12,55 |
| Study characteristics | 17 | Cite each included study and present its characteristics. | Page 12-14  Page 41-46 |
| Risk of bias in studies | 18 | Present assessments of risk of bias for each included study. | Page 14-15  Page 61-63 |
| Results of individual studies | 19 | For all outcomes, present, for each study: (a) summary statistics for each group (where appropriate) and (b) an effect estimate and its precision (e.g. confidence/credible interval), ideally using structured tables or plots. | Page 16-22 |
| Results of syntheses | 20a | For each synthesis, briefly summarise the characteristics and risk of bias among contributing studies. | Page 12-14 |
|  | 20b | Present results of all statistical syntheses conducted. If meta-analysis was done, present for each the summary estimate and its precision (e.g. confidence/credible interval) and measures of statistical heterogeneity. If comparing groups, describe the direction of the effect. | N/A |
|  | 20c | Present results of all investigations of possible causes of heterogeneity among study results. | N/A |
|  | 20d | Present results of all sensitivity analyses conducted to assess the robustness of the synthesized results. | N/A |
| Reporting biases | 21 | Present assessments of risk of bias due to missing results (arising from reporting biases) for each synthesis assessed. | N/A |
| Certainty of evidence | 22 | Present assessments of certainty (or confidence) in the body of evidence for each outcome assessed. | N/A |
| **DISCUSSION** | | |  |
| Discussion | 23a | Provide a general interpretation of the results in the context of other evidence. | Page 22-28 |
|  | 23b | Discuss any limitations of the evidence included in the review. | Page 28 |
|  | 23c | Discuss any limitations of the review processes used. | Page 28 |
|  | 23d | Discuss implications of the results for practice, policy, and future research. | Page 29-30 |
| **OTHER INFORMATION** | | |  |
| Registration and protocol | 24a | Provide registration information for the review, including register name and registration number, or state that the review was not registered. | - |
|  | 24b | Indicate where the review protocol can be accessed, or state that a protocol was not prepared. | N/A |
|  | 24c | Describe and explain any amendments to information provided at registration or in the protocol. | N/A |
| Support | 25 | Describe sources of financial or non-financial support for the review, and the role of the funders or sponsors in the review. | Page 31 |
| Competing interests | 26 | Declare any competing interests of review authors. | Page 31 |
| Availability of data, code and other materials | 27 | Report which of the following are publicly available and where they can be found template data collection forms; data extracted from included studies; data used for all analyses; analytic code; any other materials used in the review. | N/A |

**Appendix 8(b). PRISMA Abstract Checklist**

| Section and Topic | Item # | Checklist item | Reported (Yes/No) |
| --- | --- | --- | --- |
| TITLE | | |  |
| Title | 1 | Identify the report as a systematic review. | Yes |
| BACKGROUND | | |  |
| Objectives | 2 | Provide an explicit statement of the main objective(s) or question(s) the review addresses. | Yes |
| METHODS | | |  |
| Eligibility criteria | 3 | Specify the inclusion and exclusion criteria for the review. | Yes |
| Information sources | 4 | Specify the information sources (e.g. databases, registers) used to identify studies and the date when each was last searched. | Yes |
| Risk of bias | 5 | Specify the methods used to assess risk of bias in the included studies. | Yes |
| Synthesis of results | 6 | Specify the methods used to present and synthesise results. | Yes |
| RESULTS | | |  |
| Included studies | 7 | Give the total number of included studies and participants and summarise relevant characteristics of studies. | Yes |
| Synthesis of results | 8 | Present results for main outcomes, preferably indicating the number of included studies and participants for each. If meta-analysis was done, report the summary estimate and confidence/credible interval. If comparing groups, indicate the direction of the effect (i.e., which group is favoured). | Yes |
| DISCUSSION | | |  |
| Limitations of evidence | 9 | Provide a brief summary of the limitations of the evidence included in the review (e.g. study risk of bias, inconsistency and imprecision). | N/A |
| Interpretation | 10 | Provide a general interpretation of the results and important implications. | Yes |
| OTHER | | |  |
| Funding | 11 | Specify the primary source of funding for the review. | N/A |
| Registration | 12 | Provide the register name and registration number. | N/A |

*From:*  Page MJ, McKenzie JE, Bossuyt PM, Boutron I, Hoffmann TC, Mulrow CD, et al. The PRISMA 2020 statement: an updated guideline for reporting systematic reviews. BMJ 2021;372:n71. doi: 10.1136/bmj.n71

For more information, visit: <http://www.prisma-statement.org/>

**Appendix 9: Data Extraction form**

| 1. Study ID 2. Title 3. Reviewer Name 4. Title 5. Lead author contact details 6. Country in which the study conducted 7. Aim of study 8. Study design 9. Start date 10. End date 11. Study setting 12. Study funding sources 13. Possible conflicts of interest for study authors 14. Population description 15. Inclusion criteria 16. Exclusion criteria 17. Method of recruitment of participants 18. Total number of participants 19. Age of participants 20. Years diagnosed with Type 2 Diabetes Mellitus 21. Race/Ethnicity 22. Height 23. Weight 24. Sex 25. Income 26. Educational Attainment 27. Comorbidities 28. Insurance 29. Data collection method 30. Data collection Language 31. Health Literacy measure and value: 32. Health literacy Instrument 33. Health literacy Score range 34. Health literacy Value Mean +_SD 35. Health literacy % Adequate, data Limited HL 36. Health literacy Value for Ethnic minority groups 37. Medication Adherence Measure and value: 38. Medication adherence Instrument 39. Medication adherence Score range 40. Medication adherence Value Mean +_SD 41. Medication adherence % Adherent, Non-adherent 42. Medication adherence Value for Ethnic minority groups 43. Other measures and value: 44. Measure 45. Instrument |
| --- |

**References:**

1. Elsous A, Radwan M, Al-Sharif H, Abu Mustafa A. Medications Adherence and Associated Factors among Patients with Type 2 Diabetes Mellitus in the Gaza Strip, Palestine. Frontiers in Endocrinology. 2017;8:100.

2. Zeng QJ, Ying; Yuan, Yanfei; Wen, Xiuqin; Sun, Yahui; Tian, Ding; Wang, Xiaohuai; Chang, Chun. Association of health literacy with health management among diabetics. Chinese journal of preventive medicine. 2014;48(8):715-9.

3. Lee Y-J, Shin S-J, Wang R-H, Lin K-D, Lee Y-L, Wang Y-H. Pathways of empowerment perceptions, health literacy, self-efficacy, and self-care behaviors to glycemic control in patients with type 2 diabetes mellitus. Patient Education and Counseling. 2016;99(2):287-94.

4. Huang Y, Shiyanbola, O., Smith, P.,. The association of health literacy and self-efficacy with medication adherence and diabetes control. Journal of the American Pharmacists Association. 2017;57(3):745749.

5. Morris NSM, Charles D; Littenberg, Benjamin. Literacy and health outcomes: a cross-sectional study in 1002 adults with diabetes. BMC family practice. 2006;7(100967792).

6. Marmouch H, Jenzri, H., Charrada, I., Khochtali, I.,. The association of health literacy with illness perceptions, medication beliefs, and medication adherence among elderly with type 2 diabetes. Diabetes Technology & Therapeutics. 2021;23(S2):A-1-A-206.

7. Yeung RO, Belag, A., Alfaituri, M., Al-Sayah, F.,. Diabetes knowledge, self-care behaviours, and metabolic control in Arabic-speaking adults with type 2 diabetes in Edmonton, Canada. Diabetologia. 2018;61(1):1-620.

8. Krzeminska S, Lomper, K., Chudiak, A., Ausili, D., Uchmanowicz, I.,. The association of the level of self-care on adherence to treatment in patients diagnosed with type 2 diabetes. Acta diabetologica. 2021;58(4):437-45.

9. Kassahun T, Gesesew H, Mwanri L, Eshetie T. Diabetes related knowledge, self-care behaviours and adherence to medications among diabetic patients in Southwest Ethiopia: a cross-sectional survey. BMC Endocrine Disorders. 2016;16(1):28.

10. Bauer AM, Schillinger D, Parker MM, Katon W, Adler N, Adams AS, et al. Health Literacy and Antidepressant Medication Adherence Among Adults with Diabetes: The Diabetes Study of Northern California (DISTANCE). Journal of General Internal Medicine. 2013;28(9):1181-7.

11. White R, Gebretsadik T, Osborn C, Rothman R. Health literacy is associated with self-care behaviors in uninsured, low income latino adults with type 2 diabetes. Journal of General Internal Medicine. 2010;25(3):205-567.

12. Todd A, Rogers, E., King, S.,. The influence of functional health literacy on adherence to heart health medications. Journal of the American Pharmacists Association. 2015;55(2):e154.

13. Leemans C, Van den Broucke S, Philippe M-F. L’impact différentiel des dimensions de la littératie en santé sur l’adhésion au traitement des patients diabétiques. Educ Ther Patient/Ther Patient Educ. 2021;13(2):20203.

14. Shiyanbola O, Lanier, C., Huang, Y., Unni, E.,. The association of health literacy with illness perceptions, medication beliefs, self-efficacy, and medication adherence among patients with type 2 diabetes. Journal of the American Pharmacists Association. 2017;57(3):745749.

15. Osborn C, Quintero, C., Kripalani, S., Rothman, R., Sullivan, C., Crutcher, T., Pilon, B., Snell, B.,. Racial disparities in diabetes: Health literacy, numeracy, or something else? Diabetes. 2011;60:A222-A3.

16. Chin J, Wang H, Awwad AW, Graumlich JF, Wolf MS, Morrow DG. Health Literacy, Processing Capacity, Illness Knowledge, and Actionable Memory for Medication Taking in Type 2 Diabetes: Cross-Sectional Analysis. Journal of General Internal Medicine. 2021;36(7):1921-7.

17. Ilhan N, Telli, S., Temel, B., Asti, T.,. Health literacy and diabetes self-care in individuals with type 2 diabetes in Turkey. Primary Care Diabetes 2021;15(1):74-9.

18. Thurston M, Huston, S., Bourg, C., Phillips, B., Ryan, G.,. Impact of health literacy on aspects of medication nonadherence reported by underserved patients with type 2 diabetes. Pharmacotherapy: The Journal of Human Pharmacology and Drug Therapy. 2013;33(10):e182-e332.

19. Kooshyar H, Shoorvazi, M., Dalir, Z., Hosseini, M.,. Health literacy and its relationship with medical adherence and health-related quality of life in diabetic community-residing elderly. Journal of Mazandaran University of Medical Sciences. 2014;24:133-43.

20. Hur S, Curtis, L.M., Davis, T., Arnold, C., Mcsweeney, J., Jennifer, G., Kwasny, M.J., Wolf, M.S., Hadden. K.,. Modifiable factors associated with diabetes adherence in rural primary care clinics. Journal of General Internal Medicine. 2020;35(1):1-779.

21. Selvakumar D, Sivanandy P, Ingle PV, Theivasigamani K. Relationship between Treatment Burden, Health Literacy, and Medication Adherence in Older Adults Coping with Multiple Chronic Conditions. Medicina. 2023;59(8):1401.

22. Schillinger D. Validity of a Computational Linguistics-Derived Automated Health Literacy Measure Across Race/Ethnicity: Findings from The ECLIPPSE Project. Journal of Health Care for the Poor & Underserved Supplement 2021;32(2):347-65.

23. Olorunfemi O. Health literacy, medication belief, and demographic factors as correlates of medication adherence in person with diabetes mellitus. Journal of Diabetology. 2023;14(2):100-6.

24. Crespo TS, Andrade JMO, Lelis DdF, Ferreira AC, Souza JGS, Martins AMEdBL, et al. Adherence to medication, physical activity and diet among older people living with diabetes mellitus: Correlation between cognitive function and health literacy. IBRO Reports. 2020;9:132-7.

25. Ueno H, Ishikawa H, Suzuki R, Izumida Y, Ohashi Y, Yamauchi T, et al. The association between health literacy levels and patient-reported outcomes in Japanese type 2 diabetic patients. SAGE Open Medicine. 2019;7:2050312119865647.

26. Sharma D, Goel NK, Cheema YS, Garg K. Medication Adherence and its Predictors among Type 2 Diabetes Mellitus Patients: A Cross-Sectional Study. Indian Journal of Community Medicine. 2023;48(5):781-5.

27. Osborn CY, Cavanaugh K, Wallston KA, Kripalani S, Elasy TA, Rothman RL, et al. Health Literacy Explains Racial Disparities in Diabetes Medication Adherence. Journal of Health Communication. 2011;16(sup3):268-78.

28. Huang Y-M, Shiyanbola OO. Investigation of Barriers and Facilitators to Medication Adherence in Patients With Type 2 Diabetes Across Different Health Literacy Levels: An Explanatory Sequential Mixed Methods Study. Frontiers in Pharmacology. 2021;12.

29. Radwan M, Elsous A, Al-Sharif H, Abu Mustafa A. Glycemic control among primary care patients with type 2 diabetes mellitus in the Gaza Strip, Palestine. Therapeutic Advances in Endocrinology and Metabolism. 2017;9(1):3-14.

30. Rocha MRd, Santos SDd, Moura KRd, Carvalho LdS, Moura IHd, Silva ARVd. Health literacy and adherence to drug treatment of type 2 diabetes mellitus. Escola Anna Nery. 2019;23:e20180325.

31. Tefera YG, Gebresillassie BM, Emiru YK, Yilma R, Hafiz F, Akalu H, et al. Diabetic health literacy and its association with glycemic control among adult patients with type 2 diabetes mellitus attending the outpatient clinic of a university hospital in Ethiopia. PLOS ONE. 2020;15(4):e0231291.

32. Hussain N, Said ASA, Khan Z. Influence of Health Literacy on Medication Adherence Among Elderly Females With Type 2 Diabetes in Pakistan. International Quarterly of Community Health Education. 2019;41(1):35-44.

33. Ng BP, Park, C.,. Medication Non-Adherence and Health Literacy Among Medicare Beneficiaries with Type 2 Diabetes. Value in Health. 2021;24:S83.

34. Hagliroshan A. Qom University of Medical Sciences Journal. 2021;14:70-80.

35. ÖZONUK E, YILMAZ, M.,. Tip 2 Diabetes Mellitus Tanılı Hastaların Sağlık Okuryazarlığı ve Tedavi Uyumu Arasındaki İlişki. Journal of Education & Research in Nursing 2019;16(4):91.

36. Yeh J-Z, Wei C-j, Weng S-f, Tsai C-y, Shih J-h, Shih C-l, et al. Disease-specific health literacy, disease knowledge, and adherence behavior among patients with type 2 diabetes in Taiwan. BMC Public Health. 2018;18(1):1062.

37. Opsasnick L, Hur, S., Curtis, L., Batio, S., Wismer, G., Wolf, M.S.,. Comparing the performance of self-report medication adherence measures among patients with limited literacy and english proficiency. Journal of General Internal Medicine. 2019;34:171.

38. Chu SH, Choi, E.J., Kim, S.H., Nam, M.,. Health literacy, self-care activities, and glycemic control among older Korean type 2 diabetes patients. 2010;59:A280-A1.

39. Nandyala AS, Nelson LA, Lagotte AE, Osborn CY. An Analysis of Whether Health Literacy and Numeracy Are Associated with Diabetes Medication Adherence. Health literacy research and practice. 2018;2(1):e15-e20.

40. Huang Y-M, Shiyanbola OO, Chan H-Y, Smith PD. Patient factors associated with diabetes medication adherence at different health literacy levels: a cross-sectional study at a family medicine clinic. Postgraduate medicine. 2020;132(4):328-36.

41. Shiyanbola O, Unni E, Huang Y-M, Lanier C. The association of health literacy with illness perceptions, medication beliefs, and medication adherence among individuals with type 2 diabetes. RES SOC ADMIN PHARM. 2018;14(9):824-30.

42. Huang Y-M, Shiyanbola O, Smith P. Association of health literacy and medication self-efficacy with medication adherence and diabetes control. Patient preference and adherence. 2018;12:793-802.

43. Huang Y-M, Shiyanbola OO, Chan H-Y. A path model linking health literacy, medication self-efficacy, medication adherence, and glycemic control. Patient education and counseling. 2018;101(11):1906-13.

44. Osborn CY, Cavanaugh K, Wallston KA, Kripalani S, Elasy TA, Rothman RL, et al. Health literacy explains racial disparities in diabetes medication adherence. Journal of health communication. 2011;16 Suppl 3(dik, 9604100):268-78.
